# A Rare Case of Rhupus Syndrome With Systemic Involvement: A Case Report and Literature Review

**DOI:** 10.7759/cureus.32707

**Published:** 2022-12-19

**Authors:** Mohammad S Dairi, Deema S Ashoor, Ahad S Babkier, Rami Algahtani, Khaled S Dairi

**Affiliations:** 1 Department of Internal Medicine, Faculty of Medicine, Umm Al-Qura University, Makkah, SAU; 2 Department of Medicine and Surgery, Faculty of Medicine, Umm Al-Qura University, Makkah, SAU; 3 Department of Internal Medicine, King Faisal Hospital, Ministry of Health, Makkah, SAU

**Keywords:** lupus nephritis, vasculitis, autoimmune hemolytic anemia, rhupus syndrome, systemic lupus erythematosus, rheumatoid arthritis

## Abstract

Based on clinical signs, symptoms, radiological, and serological findings, a 37-year-old woman was diagnosed with an overlap between rheumatoid arthritis and systemic lupus erythematosus, referred to as rhupus syndrome. Her condition was complicated by lupus nephritis, autoimmune hemolytic anemia, and central nervous system (CNS) vasculitis. She improved after receiving steroids, hydroxyquinone, and cyclophosphamide.

There are no established criteria for diagnosing rhupus syndrome. Being aware of autoimmunity and overlapping illness signs and using specific diagnostic tests are crucial. Early therapy may avoid irreversible organ damage.

## Introduction

Rhupus is a rare clinical syndrome with features of systemic lupus erythematosus (SLE) and rheumatoid arthritis (RA), which is defined as symmetric polyarthritis that is verified to be erosive on radiographs and is accompanied by clinical SLE signs and symptoms [1-5]. Approximately, it occurs in 0.01%-2% of all cases of systemic rheumatic disease; it was first described by Peter Schur in 1971 [2,6]. A 7,000-patient epidemiological study found that the prevalence of RA was 15% and that of SLE was 8.9%. Therefore, 1.2% is the expected probability of a coincidence between RA and SLE [4]. 

A high-specificity autoantibody (anti-double-stranded DNA antibody for SLE or anti-cyclic citrullinated peptide for RA) as well as clinical signs and symptoms of SLE are present in rhupus syndrome [4,6]. Previous studies on rhupus patients revealed that RA symptoms and signs predominate over SLE-related organic damage, and the main symptom is erosive arthropathy [6,7]. However, it can manifest any of the typical SLE symptoms, including photosensitivity, serositis, vasculitis, or glomerulonephritis [6]. Unfortunately, the absence of official classification criteria places rhupus patients at a disadvantage and makes it challenging to classify them [6]. In most cases, the prognosis of rhupus syndrome is determined by the degree of the organic damage, with a higher proportion having a worse long-term prognosis [6].

In this report, we present the case of a young woman with rheumatoid arthritis and systemic lupus erythematosus overlap (rhupus) syndrome who developed central nervous system vasculitis with neuropsychiatric manifestations, autoimmune hemolytic anemia, and nephritis. To our knowledge, this is the first case report of rhupus syndrome with all these sequelae.

## Case presentation

A 37-year-old woman, whose diagnosis of rheumatoid arthritis was made eight years ago based on clinical evidence of inflammatory arthritis and serological markers of rheumatoid factor (RF) and anti-cyclic citrullinated peptide (anti-CCP), presented to the emergency department with a sudden onset of left-sided weakness and speech difficulty. Her only medication was methotrexate. On examination, she was febrile with a temperature of 39.8°C, a blood pressure of 141/100 mmHg, a regular pulse rate of 145 beats per minute, and a respiratory rate of 24 breaths per minute with an oxygen saturation of 98% on room air. Her pupils measured 3 mm on the right and 5 mm on the left, and she had a Glasgow coma scale (GCS) score of 8/15. In addition to left-sided weakness, a positive Babinski sign was also present. The remaining organ system examinations were non-revealing. Her initial investigations revealed thrombocytosis, as shown in Table 1.

**Table 1:** Patient’s initial laboratory investigations

Test	Result	Reference range
White blood cell (WBC)	9.95(x10^3/uL)	04-Nov
Red blood cell (RBC)	3.61(x10^6/uL)	3.8-4.6
Hemoglobin (HGB)	8.8 (g/dL)	Dec-15
Hematocrit (HCT)	28.8 (%)	38-46
Mean corpuscular volume (MCV)	79.8(fL)	83-101
Platelets	590(x10^9/L)	150-400

**Figure 1:**
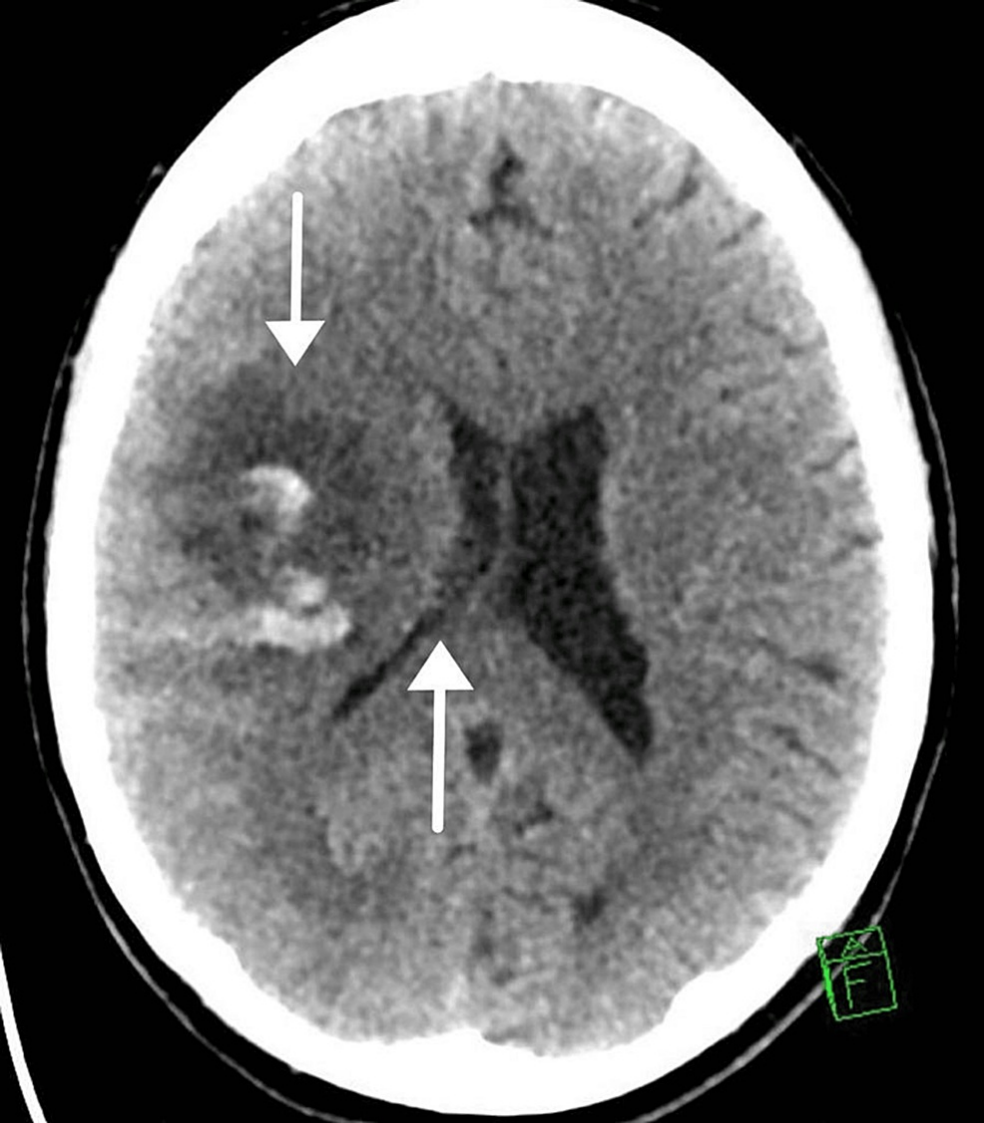
Computed tomography scan of the brain Right middle cerebral artery territory; hemorrhagic infarction associated with mass effect and midline shift

A right-sided hemorrhagic stroke with mass effect and a significant midline shift was revealed by a CT scan of the brain (Figure 1).

**Figure 2:**
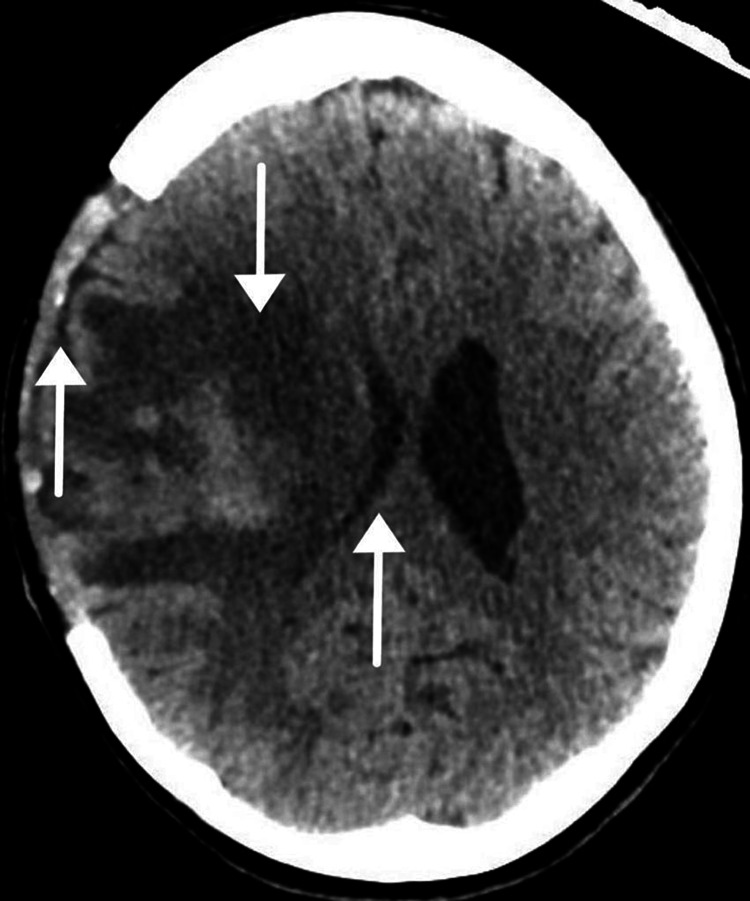
Computed tomography scan of the brain Right middle cerebral artery territory; hemorrhagic infarction post-decompressive craniectomy

She was immediately intubated and transferred to the intensive care unit (ICU) for optimization of blood pressure and osmotic therapy, in the form of mannitol and 3% hypertonic saline. She underwent an uneventful decompressive hemicolectomy and was transferred back to the ICU after an urgent neurosurgical consultation was requested. Her sedation was maintained immediately postoperatively. Her GCS improved significantly (12/15), and a post-surgery CT of the brain was performed (Figure 2).

**Figure 3:**
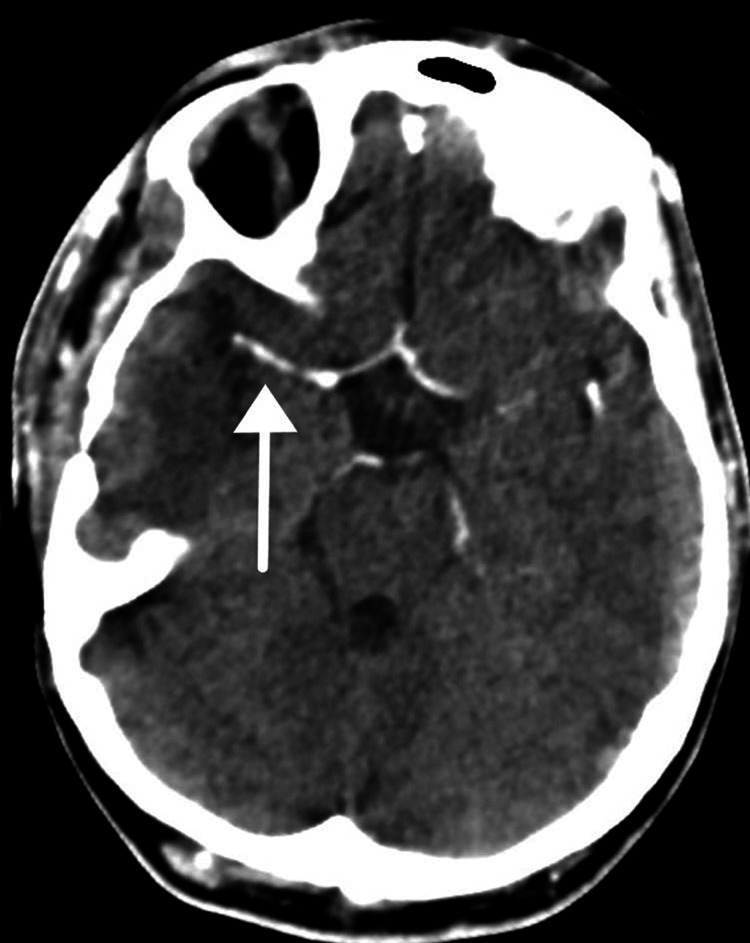
Computed tomography angiography of the brain Intracranial circulation shows irregularities and minimal to mild narrowing of the M1 segment of the right middle cerebral artery and the A1 segment of the right anterior cerebral artery.

However, her left-sided power remained at 0/5, and her aphasia persisted. On the fourth postoperative day, the patient was extubated. Further brain imaging with CT angiography was done in consultation with the neurology and rheumatology services, showing irregularities and minimal to mild narrowing of the M1 segment of the right middle cerebral artery (MCA) and the A1 segment of the right anterior cerebral artery (ACA) (Figure 3).

Additionally, her urinalysis showed evidence of proteinuria and microscopic hematuria with dysmorphic red blood cells (RBCs), and her inflammatory markers were elevated. Hence, additional autoimmune testing was sent, and it was highly consistent with the underlying new diagnosis of SLE (Table 2).

**Table 2:** Patient’s laboratory investigations

Test	Result	Reference range	Test	Result	Reference range
White blood cell (WBC)	5.87 (x10^3/uL)	04-Nov	Antinuclear antibodies (ANA)	1:1280 – homogenous pattern (titer)	>1:40 Negative
Red blood cell (RBC)	2.16 (x10^6/uL)	3.8-4.6	Anti-double stranded DNA (Anti dsDNA)	1:512 (IU/Ml)	Negative <27 Indeterminate 27-35 Positive >35
Hemoglobin (HGB)	6.3 (g/dL)	Dec-15	Immunoglobulin G (IgG)	1631.51 (mg/dL)	751-1560
Hematocrit (HCT)	19 (%)	38-46	Immunoglobulin M (IgM)	119.18 (mg/dL)	40-230
Mean corpuscular volume (MCV)	88 (fL)	83-101	Immunoglobulin A (IgA)	174.96 (mg/dL)	70-400
Mean corpuscular hemoglobin (MCH)	29.2 (PG)	27-32	C3	76.61 (mg/dL)	88-165
Mean corpuscular hemoglobin concentration (MCHC)	33.2 (g/dL)	31.5-34.5	C4	12.4 (mg/dL)	14-44
Platelets	314 (x10^9/L)	150-400	Anti-Smith Antibody (Sm)	Negative (CU)	Up To 20 Negative < 20 Positive >=20
Erythrocyte sedimentation rate (ESR)	33 (mm/Hour)	May-20	Anti-Sjögren's syndrome type B (Anti-SSB)	Negative (Units)	Up To 20 Negative < 20 Weak Positive 20 - 39 Moderate positive 40 - 80 Strong Positive> 80
C-reactive protein (CRP)	1.29 (mg/dL)	0-1	Anti-Sjögren's syndrome type A antibodies (Anti-SSA)	Negative (Units)	Up To 20 Negative < 20 Weak Positive 20 - 39 Moderate positive 40 - 80 Strong Positive> 80
Blood urea nitrogen (BUN)	15.06 (mmol/L)	2.5-6.1	Antineutrophil Cytoplasmic Autoantibody (C-ANCA)	Negative (Units)	Negative <20 Moderate Positive 20-30 Strong Positive> 30
Creatinine (CRE2)	127 (umol/L)	46-92	Perinuclear anti-neutrophil cytoplasmic antibodies (P-ANCA)	Negative (Units)	Negative <20 Moderate Positive 20-30 Strong Positive> 30
Direct Coombs test	Positive		Anti-glomerular basement membrane (Anti-GBM)	Negative (Units)	Negative = <20 Intermediate = 20-100 Positive = >100
24-hr-urine protein	7760.39 mg/day	42-225	Human immunodeficiency virus antibodies (HIV)	Negative	
Urinalysis	By direct microscope dysmorphic RBCs 10-15 RBCs/HPF		Hepatitis B virus antibodies (HBV)	Negative	
Rheumatoid factor (RF)	176 (IU/mL)	0-15	Hepatitis C virus antibodies (HCV)	Negative	
Anti-cyclic citrullinated peptide (Anti-CCP)	400 (units)	Negative = < 20 Weakly positive = 20-39 Moderately positive = 40-59 Strongly positive = >60			

Given the constellation of CNS vasculitis, autoimmune hemolytic anemia, and nephritis in the background history of RA, the diagnosis of rhupus was made, and she was given methylprednisolone pulse therapy for three days and cyclophosphamide therapy (the Euro-Lupus Protocol) of 500 mg IV every two weeks for six doses. After counseling, she refused to undergo a kidney biopsy.

The patient was discharged on oral prednisolone 60 mg once daily for 30 days, to be followed by a tapering schedule of oral hydroxyquinone 200 mg twice daily, and she is due to finish her cyclophosphamide doses in the day-care unit. Prophylactic sulfamethoxazole/trimethoprim was prescribed. A follow-up appointment with the rheumatologist, neurologist, and physiotherapist was scheduled.

## Discussion

Systemic lupus erythematosus (SLE) and rheumatoid arthritis (RA) are systemic autoimmune disorders with articular and extra-articular involvement [8,9]. Rhupus is an uncommon condition that combines SLE and RA [1-4]. Individually, SLE and RA have distinct immunopathogenic mechanisms. SLE is associated with a Th2 immune response, whereas RA is associated with a Th1 immune response. In SLE and RA, CD4+CD28-null cells are elevated, indicating a role for the senescence-associated secretory phenotype (SASP). Thus, rather than being related to a different level of immunosenescent cells, rhupus may be related to a different polarization of CD4+CD28-null cells compared to SLE [10]. Therefore, the polarization of immunosenescent T cells, the human leukocyte antigen (HLA) complex, hormonal factors, and genetic background explain how this overlap might arise among patients with rhupus syndrome [10]. Earlier studies analyzed CD4+CD28-null cells in nine rhupus and nine SLE patients and showed that they were polarized towards a Th1 phenotype in rhupus patients but partly towards a Th2 phenotype in SLE patients with non-erosive arthritis [11]. 

Rhupus is characterized by RA-like articular involvement, with no difference in the distribution of erosions and bone edema for the same parameters. It's also associated with one or more lupus-like organ involvements that are less severe compared to SLE. Rhupus patients usually have more RA-associated damage than SLE-associated damage [5,10,12]. Thus, rhupus syndrome is defined as symmetric polyarthritis that is verified to be erosive on radiographs and is accompanied by clinical SLE signs and symptoms [5]. Hematological abnormalities are the primary signs of SLE in the lupus group. Significantly more rhupus patients have leucopenia than SLE patients. Regarding the occurrence of anemia or thrombocytopenia, no significant differences were observed [5,10]. Then there are skin lesions, renal involvement, photosensitivity, serositis, oral ulcers, alopecia, Raynaud's, pulmonary involvement, neurological involvement, and vasculitis [5,10,12].

The prevalence of rheumatoid factor (RF) in rhupus patients is greater than in SLE patients, but there is no difference between rhupus and RA patients. Anti-CCP antibodies are highly specific for RA, and their prevalence in RA is higher than that in scleroderma. The prevalence of positive antinuclear (ANAs), anti-dsDNA, and anti-Smith antibodies among rhupus patients is similar to that of those with SLE, while RA patients show a lower prevalence of anti-dsDNA antibodies [10,12,13].

At diagnosis, every patient got corticosteroids and one to three disease-modifying antirheumatic drugs (DMARDs), along with hydroxychloroquine. Methotrexate, sulfasalazine, azathioprine, and leflunomide are often the prescribed DMARDs. Intravenous methylprednisolone pulses are less commonly utilized in rhupus than in SLE. Immunosuppressants, including cyclophosphamide, mycophenolate mofetil, and cyclosporin, are used to control organ damage [10]. Considering the use of biologics on rhupus patients refractory to conventional treatments, there are some worries about giving TNF-alpha inhibitors but rituximab and abatacept seem to be more promising [10,12].

In the most recent systematic review, 100 of 287 cases of rheumatoid arthritis had renal involvement, compared to 12 cases of vasculitis and two case reports of CNS vasculitis. [10,14,15] (Table 3). In the same systematic review, 202 of 287 patients were identified with hematological abnormalities, but only a few instances showed autoimmune hemolytic anemia, the majority of which had favorable outcomes [10,16-22] (Table 3).

**Table 3:** Summary of the literature ANA: antinuclear antibodies; Anti-dsDNA: anti-double-stranded DNA antibodies; Anti-CCP: anticitrullinated peptide antibodies; DCT: Direct Coombs test; TB: tuberculosis; RA: rheumatoid arthritis; CNS: central nervous system

Rhupus syndrome with CNS vasculitis									
Study	Date of publication	Place	Age and gender	Past medical history	Rhupus complications	Biomarkers	Biopsy	Treatment	Outcome
Zaman et al., 2019^14^	Oct-19	Bangladesh	34-year- old female	RA	Cerebral infarction (vasculitis suspected) Libman-Sacks endocarditis	RF ANA Anti-dsDNA Anti-CCP	No	Aspirin, hydroxychloroquine, and atorvastatin	Not mentioned
Wang et al., 2009^15^	18-06-2009	China	37-year-old female	10-year history of polyarthritis	CNS vasculitis	RF ANA Anti-dsDNA Anti-CCP	No	Methylprednisolone, phenobarbital, sodium valproate	Improved
Rhupus syndrome with autoimmune hemolytic anemia									
Ditmars et al., 2022^16^	25-05-2022	United States of America	53-year-old female	Rhupus syndrome TB	Autoimmune hemolytic anemia TB re-activation	RF Anti-dsDNA DCT	No	Steroids, rifampin, isoniazid, pyrazinamide, ethambutol	Deteriorated due to TB re-activation
Manzo & Castagna, 2021^17^	01-07-2021	Italy	38-year-old female	RA	Erosive inflammatory arthropathy Autoimmune hemolytic anemia Oral ulcers	RF ANA anti-dsDNA Anti-CCP DCT	No	Steroids, infliximab, methotrexate	Improved
Yousfi et al., 2021^18^	15-03-2021	Morocco	35-year-old male	RA	Erosive inflammatory arthropathy Autoimmune hemolytic anemia Lupus nephritis Pleuropericarditis	RF ANA Anti-dsDNA Anti-CCP DCT	Kidney biopsy	Steroids, cyclophosphamide, azathioprine	Improved
Espinosa-Orantes et al., 2020^19^	09-12-2020	Mexico	29-year-old female	Medically free	Non-erosive inflammatory arthropathy Autoimmune hemolytic anemia	ANA Anti-dsDNA Anti-CCP DCT	No	Steroids, chloroquine, azathioprine	Improved
Gutta et al., 2019^20^	19-12-2019	India	A 57-year-old female	Medically free	Erosive inflammatory arthropathy Organising pneumonia Autoimmune hemolytic anemia Oral ulcers	RF ANA DCT	Transbronchial lung biopsy, Left axillary lymph node biopsy	Hydroxychloroquine, Steroids, sulphasalzine, azathioprine	Improved
Sinha et al., 2019^21^	14-01-2019	India	55‑year‑old female	Medically free	Erosive inflammatory arthropathy skin lesions	RF Anti-CCP DCT	Skin biopsy	Steroids, hydroxychloroquine, Methotrexate	Improved
Min et al., 2014^22^	30-12-2014	Korea	45-year-old female	Medically free	Erosive inflammatory arthropathy Autoimmune hemolytic anemia	RF ANA Anti-dsDNA Anti-CCP DCT	No	Steroids, hydroxychloroquine, celecoxib, methotrexate	Improved

Our patient was diagnosed with an overlap between RA and SLE based on clinical signs and symptoms and serological findings, and her illness was complicated by lupus nephritis, autoimmune hemolytic anemia, and CNS vasculitis, which improved following administration of steroids, hydroxyquinone, and cyclophosphamide.

As rhupus is an uncommon disorder and a delay in diagnosis may result in deformities, other organ issues, or death, physicians must be alert for autoimmunity and overlapping features. To confirm a rhupus diagnosis, specific diagnostic tests are essential.

Despite the increasing number of case reports and series, there is still no consensus on the diagnostic criteria for rhupus syndrome. In all the descriptive studies, including case reports, association does not suggest a cause-and-effect link, and their findings cannot be generalized. To further comprehend this sequential, dynamic overlap syndrome and to characterize its clinical and immunologic phenotype, we encourage extensive prospective studies monitoring patients from the onset of symptoms to an extended follow-up period.

## Conclusions

Rhupus is a rare syndrome that combines SLE and RA, with greater end-organ damage than RA alone. There are currently no established criteria for diagnosing rheumatoid arthritis. Clinical, serological, and imaging findings can play a significant role in confirming the diagnosis. It is usually treated with corticosteroids and one to three DMARDs, with biologics considered in refractory cases. Early treatment can prevent the occurrence of irreversible organ damage.
